# Extreme prematurity and attention deficit: epidemiology and prevention

**DOI:** 10.3389/fnhum.2013.00578

**Published:** 2013-09-19

**Authors:** T. Michael O'Shea, L. Corbin Downey, Karl K. C. Kuban

**Affiliations:** ^1^Division of Neonatology, Department of Pediatrics, Wake Forest School of Medicine, Winston-SalemNC, USA; ^2^Division of Pediatric Neurology, Department of Pediatrics, Boston UniversityBoston, MA, USA

**Keywords:** extreme prematurity, neurodevelopmental impairment, attention, attention deficit hyperactivity disorder, neuroprotection

## Extreme prematurity and attention impairment

Preterm infants are at increased risk for a wide range of developmental disorders, including sensory, motor, cognitive, and other brain disorders (Lorenz et al., 1998; Bhutta et al., 2002; Aarnoudse-Moens et al., 2009), and the risk is highest for those infants born before 28 weeks gestation, i.e., extremely preterm or extremely low gestational age infants (Wood et al., 2005; Serenius et al., 2013). As large cohorts of extremely preterm infants have reached school age, the prevalence of brain dysfunctions that affect academic success has been quantified, and antecedents and correlates of these problems have been better characterized. The most prevalent of these is attention deficit/hyperactivity disorder (ADHD) (Hack et al., 2009; Johnson et al., 2010).

Based on screening questionnaires, such as the Child Behavioral Checklist (Hille et al., 2001) and the Strengths and Difficulties Questionnaire (Elgen et al., 2002; Samara et al., 2008; Delobel-Ayoub et al., 2009), children born extremely preterm perform worse than full term children on attention scales. Using Diagnostic and Statistical Manual-based criteria, extremely preterm children have a risk of ADHD that is four times that of full term controls (Johnson et al., 2010; Scott et al., 2012).

Some studies report an association of extreme prematurity with the inattention type of ADHD but not the hyperactivity/impulsivity type (Hack et al., 2009; Johnson et al., 2010; Johnson and Marlow, 2011), while others report associations with both types of ADHD (Anderson et al., 2011; Scott et al., 2012). In one sample, inattentive behaviors were explained by sequential memory problems, while hyperactive behaviors were explained by global intellectual impairment (Nadeau et al., 2001). The attention impairment among preterm infants affects a range of domains of attention including selective attention, sustained attention, attention encoding, shifting attention, and divided attention (Mulder et al., 2009; Anderson et al., 2011).

In the general population ADHD is associated with conduct disorder (Nock et al., 2006), but this does not appear to be the case among preterm infants (Elgen et al., 2002; Hack et al., 2009; Johnson et al., 2010; Scott et al., 2012). Extremely preterm infants with ADHD are more likely to have cognitive impairment than those without ADHD, and in one study there was no association between extreme prematurity and ADHD among infants without cognitive impairment (Johnson et al., 2010). Impaired attention is a likely contributor to extremely preterm children's increased risk of cognitive impairment and behavioral problems (Weijer-Bergsma et al., 2008). Moderately preterm children exhibit some developmental catch up in selective attention so that the difference between these children and term children narrows with increasing age (Mulder et al., 2009).

## Risk factors for attention impairment among extremely preterm infants

Social disadvantage is more prevalent among mothers delivering prematurely (Paneth, 1995), and is a risk factor for attention problems during childhood among preterm infants (Hack et al., 2009; Lindstrom et al., 2011; Scott et al., 2012). This variable conveys information about a variety of factors including race, maternal psychosocial stress, and mother's education (Adler et al., 2012). In unselected samples, maternal smoking, which is associated with preterm delivery, has been associated with attention impairment (Nomura et al., 2010).

The strong inherited contribution to ADHD (Thapar et al., 2012) appears to be less important among preterm infants (Johnson and Marlow, 2011). Male sex, which is predictive of more severe neonatal illness after preterm birth, is associated with the hyperactive type of ADHD among extremely low birth weight children (Hack et al., 2009). Neonatal illnesses which occur frequently after extremely preterm birth, such as necrotizing enterocolitis and chronic lung disease, could explain the smaller contribution of genetics in this group. In one extremely preterm cohort, necrotizing enterocolitis was predictive of impaired selective attention but not other attention domains (Anderson et al., 2011). At school age, children who had recovered from neonatal chronic lung disease, as compared to preterm children without chronic lung disease, had more attention problems, based on teacher's report (Gray et al., 2008). However, in two other cohorts no neonatal factors were predictive of an attention problem (Hack et al., 2009; Johnson et al., 2010). In another cohort of extremely preterm children, an Apgar score less than 8 at 5 min was associated with a higher risk of using medication for ADHD (Lindstrom et al., 2011).

Among very low birth weight infants, intraventricular hemorrhage (and presumably the accompanying brain damage) (Indredavik et al., 2010) and subnormal head growth (Peterson et al., 2006) are associated with attention problems. In a large prospective study, white matter injury was associated with a 2.7-fold increase in the risk of ADHD at 6 years of age (Whitaker et al., 1997). Ultrasound is only modestly sensitive for detection of white matter abnormalities (Maalouf et al., 2001; Inder et al., 2003; Miller et al., 2003). More sensitive imaging techniques, using magnetic resonance imaging (MRI) also have identified structural correlates of attention impairment. Among adolescents who had very low birth weight, thinning of the corpus callosum and reduced white matter volume were associated with attention deficit but were not associated with hyperactivity (Indredavik et al., 2005). Diffuse tensor imaging, which identifies disruption or disorganization of white matter tracts, indicates that reduced fractional anisotropy of the external capsule and middle and superior fascicles is associated with higher inattention scores on the ADHD Rating Scale IV (Skranes et al., 2007).

## Inflammation and cerebral white matter damage in extremely preterm infant

Even when an infection is distant from the brain, maternal and neonatal infections are associated with perinatal brain damage (Dammann and O'Shea, 2008). Administration of endotoxin to a variety of immature experimental animals results in cerebral damage, and the damage is mediated by inflammation-related molecules including cytokines, chemokines, adhesion molecules, and matrix metalloproteinases (Wang et al., 2006). A range of clinical disorders in humans has been associated with perinatal infection and inflammation, including ultrasound-defined white matter injury, microcephaly, cerebral palsy, cognitive impairment, behavioral dysfunctions, and psychiatric illness (Hagberg et al., 2012).

Biomarkers of perinatal infection and inflammation include neutrophil infiltration of the placenta (Holzman et al., 2007) and inflammation-related proteins in the amniotic fluid and neonatal blood. Clinical initiators of inflammation include maternal infections (McElrath et al., 2011), lung injury induced by mechanical ventilation (Bose et al., 2013), necrotizing enterocolitis (Martin et al., 2013), and neonatal sepsis (Leviton et al., 2012).

In a large cohort of extremely preterm infants, the ELGAN cohort, both clinical indicators (McElrath et al., 2009; Martin et al., 2010) and biomarkers of inflammation (Leviton et al., 2010) have been associated with perinatal brain damage and subsequent developmental impairment at 2 years of age. In this cohort, persistent/recurrent elevations of seven inflammation-related proteins, defined as an elevation on at least 2 days a week or more apart in the first 2 weeks of life, are associated with a 2- to 3.9-fold increase in the risk of an attention impairment identified at 2 years of age using the Child Behavioral Checklist [manuscript under review].

Maternal or neonatal infections occur in a majority of pregnancies that result in an extremely preterm birth, yet the prevalence of ADHD among the offspring is typically less than 20%, suggesting that inflammation requires other factors, which could include genetic susceptibility, to contribute to the occurrence of ADHD. In a genetically isolated community with a high prevalence of ADHD, severe maternal respiratory infection was associated with a 3.3-fold increase in risk, suggesting that genetic factors could modify associations between inflammation and ADHD in humans (Pineda et al., 2007). In a preclinical model, inflammation-induced attentional impairments and abnormalities in dopamine neurons were more severe in mice genetically deficient in Nurr1, which plays important roles in differentiation, migration, and survival of dopaminergic neurons (Vuillermot et al., 2012).

## Might interventions to reduce perinatal inflammation decrease the risk of attention impairments among extremely preterm children?

### Antenatal interventions

The consistent association of perinatal inflammation and brain disorders, including attention impairment, suggests that immuno-modulatory interventions might decrease the risk of attention problems in extremely preterm infants.

Antenatal treatment of the mother with glucocorticoids might modulate inflammation's effects on the brain. For example, antenatal glucocorticoids decrease the risk of cerebral palsy (Roberts and Dalziel, 2006). However, in two randomized clinical trials of antenatal steroids, attention abilities were not improved, nor was the risk of ADHD reduced, by this intervention (Dalziel et al., 2005; Crowther et al., 2007).

Maternal infection is a frequent initiator of preterm labor (Romero et al., 2007), and often is accompanied by a fetal systemic inflammatory response (Gotsch et al., 2007). However, antenatal antibiotic treatment of mothers with preterm labor, but without overt infection, does not decrease the risk of attention problems in the offspring (Kenyon et al., 2008a,b).

Antenatal treatment with magnesium sulfate reduces the risk of cerebral palsy in offspring of mothers who develop preterm labor prior to 30 weeks gestation (Rouse, 2007). However, the effect of this intervention on attention problems has not been reported (Doyle et al., 2009).

Children of obese mothers are more likely than children of women with a pre-pregnancy weight in the normal range to have a low Bayley Scales Mental Development Index at age 2 years (Hinkle et al., 2012) and a lower reading score at kindergarten age (Hinkle et al., 2013). Since maternal pre-pregnancy obesity is associated with later inflammation in the offspring (Leibowitz et al., 2012), interventions that reduce maternal obesity could reduce the risk of attention problems in the offspring.

### Postnatal interventions

Postnatal strategies to decrease inflammation-related perinatal brain injury include interventions to prevent initiators of inflammation and broader strategies to modulate inflammation.

The three most obvious initiators of systemic inflammation are bacteremia (Leviton et al., 2012), mechanical ventilation. (Bose et al., 2013), and necrotizing enterocolitis (Martin et al., 2013). Our hope is that whatever reduces the occurrence of these three major complications in the NICU will reduce the later occurrence of attention problems.

Broader strategies to modulate inflammation include those that shorten or minimize the intensity of inflammation once initiated. For example, caffeine reduces the risk of chronic lung disease, an inflammatory pulmonary condition, and decreases the risk of neurodevelopmental impairment. Unfortunately, the effects of perinatal caffeine on attention problems have not been reported (Schmidt et al., 2007).

Although postnatal steroids decrease lung inflammation (Halliday et al., 2009, 2010), no evidence has been offered to date that attention abilities are improved by postnatal steroids (Yeh et al., 2004). Similarly, human milk is associated with a reduced risk of necrotizing enterocolitis (Sisk et al., 2007), but other than a small pilot randomized trial of sphingomyelin-fortified human milk (Tanaka et al., 2013), evidence is lacking of an effect of human milk on attention in extremely preterm infants.

Other potential approaches to broadly reduce systemic inflammation have been suggested by preclinical studies. In animal models of perinatal brain injury which either directly or indirectly involve inflammation, (Hagberg et al., 2002; Wang et al., 2006, 2009; Thornton et al., 2012) injury can be attenuated by hypothermia (Fukuda et al., 2001; Tomimatsu et al., 2001, 2003), melatonin (Robertson et al., 2013), pentoxifylline (a methyl xanthine) (Dilek et al., 2013), and erythropoietin (Kumral et al., 2007). Hypothermia is an effective neuroprotective agent in humans born near term (Jacobs et al., 2013), and will be studied in preterm infants [ClinicalTrials.gov identifier: NCT01793129]. Melatonin and erythropoietin also are being studied as neuroprotective strategies for preterm infants [ClinicalTrials.gov identifier: NCT00649961 (melatonin) and NCT01378273 (erythropoietin)]. As mentioned above, caffeine, a methyl xanthine, appears to be neuroprotective in preterm infants although data about it effect on attention is lacking.

In addition to acute interventions, strategies might be found for attenuating the sustained disruption to brain development that persists months and perhaps years after an initial insult to the immature brain. The mechanisms underlying sustained disruption appear to include sustained inflammation as well as epigenetic changes, in which case an extended window of opportunity for intervention might exist (Fleiss and Gressens, 2012).

## Summary

Extremely preterm infants have an increased risk of attention problems and a better understanding of the antecedents of these problems can lead to prevention strategies. Perinatal systemic inflammation, an antecedent of structural and functional brain disorders in extremely preterm infants, appears to be an antecedent of attention problems. Interventions to prevent initiators of inflammation or modulate systemic inflammation might decrease the risk of attention problems among children born extremely preterm.

## Author contribution

T. Michael O'Shea wrote the initial draft of the paper. L. Corbin Downey and Karl K. C. Kuban revised the paper. All authors approved the final version.
